# Factors Affecting Outcomes in Patients With Stage III & IV Squamous Cell Carcinoma of Oropharynx: The Importance of p16 Status, BMI, and Race

**DOI:** 10.7759/cureus.13674

**Published:** 2021-03-03

**Authors:** Mary R Nittala, Madhava R Kanakamedala, Eswar Mundra, Toms Vengaloor Thomas, Eldrin Bhanat, William C Woods, Srinivasan Vijayakumar

**Affiliations:** 1 Radiation Oncology, University of Mississippi Medical Center, Jackson, USA; 2 Radiation Oncology, Texas Oncology, Amarillo, USA; 3 Orthopaedic Surgery, University of Mississippi Medical Center, Jackson, USA

**Keywords:** oropharyngeal carcinoma, racial disparities, hpv/p16

## Abstract

Objective

To identify racial disparities in survival outcomes among Stage III & IV patients with squamous cell carcinomas (SCCa) of the oropharynx treated with definitive radiation therapy (RT), with concurrent chemotherapy.

Method

This is a retrospective analysis of patients with stage III & IV SCCa of oropharynx treated with definitive RT at the State Academic Medical Center. All patients were treated to 70 Gy utilizing intensity-modulated radiation treatment (IMRT), and received concurrent chemotherapy with weekly cisplatin or cetuximab. Chi-square test was used to test the goodness of fit, overall survival (OS), and locoregional control (LRC) comparing races were generated by using Log-rank test & Kaplan-Meier method. The covariables associated with the OS and LRC were determined by the Cox regression model. A p-value of less than 0.05 was considered statistically significant. The SPSS 24.0 software (IBM Corp., Armonk, NY) was used.

Results

In the total 73 eligible patients, 54.8% were black, and 45.2% white patients. Stage distribution (per American Joint Committee on Cancer-AJCC 8^th ^Ed) between black patients vs. white patients, Stage III (45.5% vs. 54.5%) and for Stage IV (56.5% vs*.* 43.5%); p=0.499. Median follow-up for the entire group was 41 months (range: 4-144 months). In the univariate analysis, variables p16 status, body mass index (BMI), alcohol history and tumor subsite were found to be significant. In the multivariate analysis, only BMI has shown to be significant.

Three-year LRC for black patients was 37.8% vs.66.8% in white patients (p=0.354) and three-year OS for black patients was 51.8% vs. 80.9% for white patients (p*=*0.063), respectively. Five-year OS for p16 positive patients was 69.7% vs. 43% for p16 negative patients (p=0.034). Five-year OS for Stage IV black patients was 34% vs. 69.5% for Stage IV white patients (p*=*0.014).

Conclusion

Among all the co-variables examined, only BMI has shown affecting the OS outcomes; gender and BMI shown to be affecting the LRC. Racial factor appears to be significant in Stage IV patients.

## Introduction

Oropharyngeal squamous cell carcinomas (SCCa) include cancers of the base of the tongue, the palatine tonsils, the soft palate, and the posterior oropharyngeal wall [1], and it is the sixth most commonly diagnosed cancers in the United States [2], accounting for 3% of all new cases of cancer in the United States [3]. In previous studies, the increased incidences of oropharyngeal cancers has been strongly associated with tobacco and alcohol exposure but it’s been noted that incidence of oropharyngeal SCCa is high among middle-aged nonsmokers or light smokers male whites compared with older men with a significant history of drinking and smoking [4-7]. Approximately 75% of newly diagnosed oropharyngeal SCCa cases are related to human papillomavirus (HPV) [8,9]. According to the Center for Disease Control and Prevention (CDC), each year about 14,800 new HPV/p16- related oropharyngeal SCCa cases are diagnosed in men and 3,400 in women [10]. About 8.8 men and 1.8 women per 100,000 were diagnosed with HPV/p16- related oropharyngeal SCCa among white people, and 6.6 men and 1.4 women per 100,000 among black people [10]. Black people are less likely to have HPV/p16-related oropharyngeal SCCa therefore, black patients have shown to be associated with poor overall survival (OS) when compared to white patients [11,12]. The racial disparities among oropharyngeal cancers reflect the interplay of age, gender, socioeconomic factors, culture, diet, stress, state of residence, the environment and biology [13,14]. 

The aim of this study is to investigate the OS and locoregional control (LRC) of advanced-stage oropharyngeal SCCa treated at the University of Mississippi Medical Center (UMMC), and whether these differences are related to patient race, tumor characteristics and or socioeconomic status (SES).

This article was previously presented as a scientific meeting abstract at the 2019 AACR-AHNS Head and Neck Cancer Conference at Austin, TX, USA on April 29, 2019 (DOI: 10.1158/1557-3265.AACRAHNS19-B21).

## Materials and methods

Patients

An institutional review board (IRB # 2010-0252) approval was obtained for retrospective analysis of oropharyngeal SCCa patients treated by definitive RT, with concurrent chemotherapy from 2011 to 2018 at UMMC, Jackson, MS, USA. We used the browser-based database tool Research electronic data capture (RedCap) to gather and store the patient's information in password-protected computers. All the patient’s details for demographics, disease presentation, disease staging, treatments, and complications were recorded at the time of their enrollment in the database. Patient identifiers were removed before extracting the data. All the patient’s histology is identified as SCCa by biopsy and selected only Stages III & IV. The variables race, gender, smoking and alcohol history were self-reported. The variables weight, body mass index (BMI) were measured before and after treatment. All the demographic variables, disease presentation, disease staging (AJCC stage 8th edition), treatments, and complications for each patient were recorded. Sample size (n=73) with 80% power to detect the factors affecting the survival in this study was estimated by using sample size calculator. 

Treatment methods

All patients were treated to 70 Gy utilizing intensity-modulated radiation treatment (IMRT), and all patients received concurrent chemotherapy with weekly cisplatin, 40mg/m^2^ (61.6%) or weekly cetuximab, 400 mg/m^2^ (38.4%). All the treatment plans were evaluated by departmental peer review before the initiation [15]. HPV infection was confirmed to be positive, if 70% or more of the tumor cells show cytoplasmic and nuclear staining for p16 [16,17].

Statistical analysis

Fisher test is used to evaluate the racial comparison between black patients and white patients. OS for all the 73 patients in the cohort were calculated from the date of disease diagnosis and the patients last contact date. To evaluate the OS and LRC, Kaplan-Meier method was used and the comparison between black patients & white patients was measured by the log-rank test. The covariables associated with the OS and LRC were determined by the univariate and multivariate Cox regression model. Hazards ratio (HR) were estimated by time to event outcome with associated 95% confidence intervals (CIs) and p-values ≤ 0.05 were considered significant. The SPSS 24.0 software (IBM Corp., Armonk, NY) was used for data analysis.

## Results

Patient characteristics

**Figure 1:**
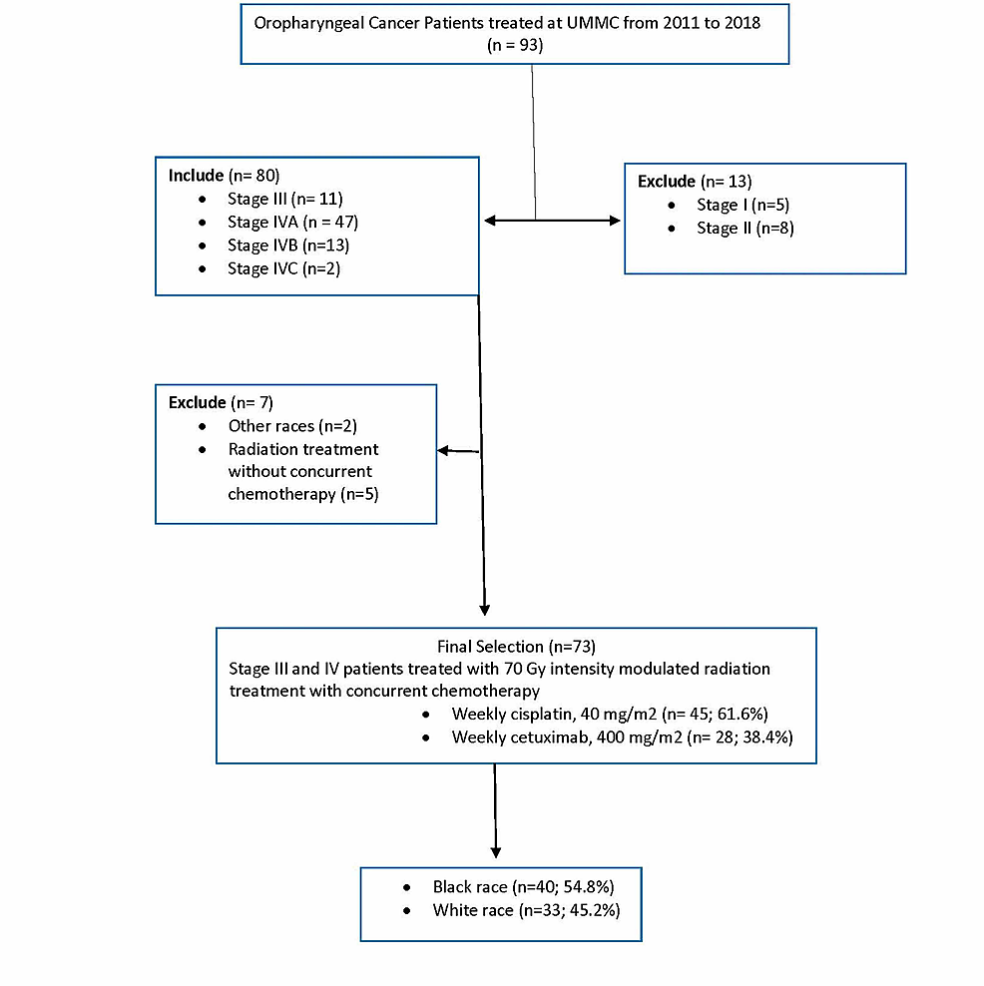
Consort diagram of patients included. UMMC: University of Mississippi Medical Center.

This study includes a total of 73 patients with SCCa of the oropharynx treated from 2011 to 2018 at UMMC. The median follow-up was 41 months (range: 4 to 144 months). Among the total 73 eligible patients, 54.8% were black patients, and 45.2% were white patients (Figure 1). Summary of patient’s demographic and clinical description was represented in (Table 1). There was no significant difference in gender or sex, age, BMI, employment status, distance traveled to the treatment facility, marital status smoking and alcohol history between black patients and white patients.

**Table 1:** Demographic and clinical characteristics of the study population by race. BMI: body mass index; BOT: base of tongue; HPV: human papillomavirus; n: number.

	Black race (n = 40; 54.8%)	White race (n = 33; 45.2%)	All patients (n = 73; 100%)	p-value
Gender/sex				
Male	30 (75.0%)	28 (84.8%)	58 (79.5%)	0.300
Female	10 (25.0%)	5 (6.8%)	15 (20.5%)	
Age				
< 50 years	6 (15.0%)	3 (9.1%)	9 (12.3%)	0.445
> 50 years	34 (85.0%)	30 (90.9%)	64 (87.7%)	
BMI				
Underweight (under 18.5 kg/m^2^)	18 (45.0%)	8 (24.2%)	16 (21.9%)	0.223
Normal (18.5 to 24.9 kg/m^2^)	9 (22.5%)	7 (21.2%)	26 (35.6%)	
Overweight (25.0 to 29.9 kg/m^2^)	5 (12.5%)	8 (24.2%)	13 (17.8%)	
Obese (over 30.0 kg/m^2^)	8 (20.0%)	10 (30.3%)	18 (24.7%)	
Income quartile				
< $ 35000 - Low Income	25 (62.5%)	12 (36.4%)	37 (50.7%)	0.026
> 35,000	15 (37.5%)	21 (63.6%)	36 (49.3%)	
Insurance				
Medicaid	22 (55.0%)	10 (30.3%)	32 (43.8%)	0.072
Medicare	9 (22.5%)	9 (27.3%)	18 (24.7%)	
Private	6 (15.0%)	5 (15.2%)	11 (15.1%)	
Self-pay	3 (7.5%)	9 (27.3%)	12 (16.4%)	
Employment				
Employed	12 (30.0%)	9 (27.3%)	21 (28.8%)	0.798
Unemployed	28 (70.0%)	24 (72.7%)	52 (71.2%)	
Distance from facility				
< 25 miles	7 (17.5%)	8 (24.2%)	15 (20.5%)	0.147
26-50 miles	7 (17.5%)	3 (9.1%)	10 (13.7%)	
51-100 miles	17 (42.5%)	8 (24.2%)	25 (34.2%)	
> 100 miles	9 (22.5%)	14 (42.4%)	23 (31.5%)	
Smoking status				
Smoker	37 (92.5%)	29 (87.9%)	66 (90.4%)	0.505
Non-smoker	3 (7.5%)	4 (12.1%)	7 (9.6%)	
Alcohol status				
Drinker	29 (72.5%)	21 (63.6%)	50 (68.5%)	0.417
Non-drinker	11 (27.5%)	12 (36.4%)	23 (31.5%)	
Marital status				
Single	25 (62.5%)	19 (57.6%)	44 (60.3%)	0.669
Married	15 (34.2%)	14 (42.4%)	29 (39.7%)	
HPV/p16 status				
Negative	32 (80.0%)	17 (51.5%)	49 (67.1%)	0.010
Positive	8 (20.0%)	16 (48.5%)	24 (32.9%)	
Subsite				
Posterior oropharyngeal wall	5 (12.5%)	3 (9.1%)	8 (11.0%)	0.425
BOT	4 (10.0%)	1 (3.0%)	5 (6.8%)	
Palatine tonsil	31 (77.5%)	29 (87.9%)	60 (82.2%)	
T stage				
T0	0 (0.0%)	0 (0.0%)	0 (0.0%)	N/A
T1	3 (7.5%)	9 (27.3%)	12 (16.4%)	0.023
T2	16 (40.0%)	12 (36.4%)	28 (38.4%)	0.750
T3	5 (12.5%)	5 (15.2%)	10 (13.7%)	0.743
T4	16 (21.9%)	6 (8.2%)	22 (30.1%)	0.043
N stage				
N0	5 (12.5%)	3 (9.1%)	8 (11.0%)	0.643
N1	2 (5.0%)	5 (15.2%)	7 (9.6%)	0.143
N2	27 (67.5%)	22 (66.7%)	49 (67.1%)	0.940
N3	6 (15.0%)	2 (6.1%)	8 (11.0%)	0.224
M stage				
M0	38 (95.0%)	32 (97.0%)	70 (95.9%)	0.673
M1	2 (5.0%)	0 (0.0%)	2 (2.7%)	0.386
Unknown	0 (0.0%)	1 (3.0%)	1 (1.4%)	N/A
Overall stage				
III	5 (12.5%)	6 (18.2%)	11 (15.1%)	0.499
IV	35 (87.5%)	27 (81.8%)	62 (84.9%)	

A significant relationship between income quartile (p=0.026), and p16 status (p=0.010) was identified among different races (Table 1); however, there were no racial differences in tumor location and cancer stage at the time of diagnosis. Regarding the income quartile, where both the races showed statistically significant (p=0.026), the majority of black patients (62.5%) belong to lower-income quartile and the majority of white patients to higher-income quartile (63.6%). For the p16 status (p=0.010), black patients showed a majority of p16 negative (80.0%) compared to white patients (51.5%), and white patients showed 48.5% of p16 positive compared to 20% among black patients.

Cox regression analysis for overall survival 

In the univariate OS Cox regression analysis, variables like BMI, p16 status, alcohol history, and tumor subsites showed a statistically significant difference in disease prognosis (Table 2). 

**Table 2:** Univariable and multivariable Cox regression for overall survival. BMI: body mass index; BOT: base of tongue; HPV: human papillomavirus; CI: confidence interval.

	Univariable		Multivariable	
	HR (95 % CI)	p-value	HR (95% CI)	p-value
Gender/sex				
Male	1		_	
Female	1.32 (0.54-3.21)	0.541	_	_
Age				
< 50 years	1		_	
> 50 years	1.06 (0.37-3.03)	0.906	_	_
Ethnicity				
Black race	1		_	
White race	1.93 (0.95-3.94)	0.069	_	_
BMI				
Normal (18.5 to 25 kg/m^2^)	1		1	
Underweight (under 18.5 kg/m^2^)	3.18 (0.84-12.04)	0.088	2.50 (0.63-9.91)	0.192
Overweight (25.0 to 29.9 kg/m^2^)	6.72 (1.97-22.88)	0.002	4.75 (1.31-17.24)	0.018
Obese (over 30.0 kg/m^2^)	1.35 (0.27-6.70)	0.711	1.24 (0.24-6.21)	0.792
Income quartile				
< $ 35,000 - low income	1		_	
> 35,000	1.08 (0.54-2.15)	0.815	_	_
Insurance				
Medicaid	1		_	
Medicare	1.81 (0.60-5.44)	0.288	_	_
Private	1.56 (0.49-4.92)	0.448	_	_
Self-pay	0.86 (0.19-3.85)	0.845	_	_
Employment				
Employed	1		_	
Unemployed	0.43 (0.17-1.04)	0.064	_	_
Distance from facility				
< 25 miles	1		_	
26-50 miles	0.50 (0.17-1.45)	0.204	_	_
51-100 miles	1.35 (0.49-3.67)	0.557	_	_
> 100 miles	0.85 (0.37-1.94)	0.706	_	_
Smoking status				
Yes	1		_	
No	0.04 (0.00-3.57)	0.176	_	_
Alcohol status				
Yes	1		_	
No	0.37 (0.14-0.96)	0.042	0.38 (0.14-1.03)	0.059
Marital status				
Single	1		_	
Married	1.01 (0.50-2.03)	0.974	_	_
HPV/p16 status				
Negative	1		1	
Positive	0.41 (0.18-0.96)	0.040	0.65 (0.26-1.62)	0.362
Subsite				
Posterior oropharyngeal wall	1		1	
BOT	2.91 (1.18-7.16)	0.020	1.96 (0.74-5.13)	0.171
Palatine tonsil	1.00 (0.23-4.25)	1.000	0.78 (0.18-3.37)	0.746
Overall stage				
III	1		_	_
IV	0.63 (0.22-1.79)	0.388	_	_

Regarding BMI, the overweight patients (25.0 to 29.9 kg/m^2^) presented six times the risk of death (HR 6.72, 95% CI, 1.97-22.88; p=0.002), and the patients with no alcohol history presented a 63% decreased risk of death (HR 0.37, 95% CI, 0.14-0.96; p=0.042); neither income quartile nor the health insurance showed any statistical significance. Variables like employment status, distance traveled to the facility, smoking status, and marital status did not show any significance statistically.

In the univariate analysis considering the tumor characteristics, p16 status and tumor subsites showed statistical significance in the prognosis. The p16 positive patients showed 59% decreased risk of mortality (HR 0.41, 95% CI, 0.18-0.96; p=0.040) compared to p16 negative patients (Table 2). The base of tongue (BOT) presented twice the risk of dying from oropharyngeal SCCa (HR 2.91, 95% CI, 1.18-7.16; p=0.020) compared to posterior oropharyngeal wall and palatine tonsils. Overall tumor staging did not significantly influence the overall survival of the patients (Table 2).

The multivariate Cox regression was built in which all the significant variables, BMI, p16 status, alcohol history, and tumor subsites were added in the model. It is interesting to notice that BMI is the only variable that showed the statistical significance and the risk is reduced 30% in the multivariate model (HR 4.75, 95% CI, 1.31-17.24; p=0.018). The patients with overweight BMI (25.0 to 29.9 kg/m^2^) showed almost five times the risk of dying from oropharyngeal SCCa (HR 4.75, 95% CI, 1.31-17.24; p=0.018) (Table 2).

Cox regression analysis for locoregional control

In the univariate Cox regression analysis for LRC, variables like gender, and BMI showed a statistically significant difference in disease prognosis. When analyzed by gender, female demonstrated twice the risk of death from the oropharyngeal cancer compared to males (HR 2.08, 95% CI, 1.03-4.23; p=0.041). Regarding BMI, the overweight patients (25.0 to 29.9 kg/m^2^) presented twice the risk of relapsing oropharyngeal SCCa (HR 1.98, 95% CI, 1.01-3.90; p=0.047) (Table 3).

**Table 3:** Univariable and multivariable Cox regression for locoregional control. BMI: body mass index; BOT: base of tongue; HPV: human papillomavirus; CI: confidence interval.

	Univariable		Multivariable	
	HR (95% CI)	p-value	HR (95% CI)	p-value
Gender/sex				
Male	1		1	
Female	2.08 (1.03-4.23)	0.041	2.65 (1.25-5.60)	0.010
Age				
< 50 years	1		_	
> 50 years	1.04 (0.49-2.21)	0.901	_	_
Ethnicity				
Black race	1		_	
White race	1.24 (0.74-2.07)	0.410	_	_
BMI				
Normal (18.5 to 24.9 kg/m^2^)	1		1	
Underweight (under 18.5 kg/m^2^)	1.10 (0.52-2.32)	0.788	1.31 (0.61-2.81)	0.476
Overweight (25.0 to 29.9 kg/m^2^)	1.98 (1.01-3.90)	0.047	2.76 (1.32-5.76)	0.007
Obese (over 30.0 kg/m^2^)	0.80 (0.37-1.72)	0.580	0.98 (0.45-2.12)	0.969
Income quartile				
< $ 35,000 - low income	1		_	
> 35,000	1.02 (0.61-1.69)	0.934	_	_
Insurance				
Medicaid	1		_	
Medicare	1.18 (0.55-2.52)	0.653	_	_
Private	0.50 (0.21-1.19)	0.12	_	_
Self-pay	0.78 (0.29-2.05)	0.621	_	_
Employment				
Employed	1		_	
Unemployed	0.58 (0.32-1/05)	0.073	_	_
Distance from facility				
< 25 miles	1		_	
26-50 miles	0.56 (0.26-1.18)	0.128	_	_
51-100 miles	1.64 (0.72-3.72)	0.232	_	_
> 100 miles	0.73 (0.39-1.37)	0.339	_	_
Smoking Status				
Yes	1		_	
No	0.73 (0.31-1.71)	0.476	_	_
Alcohol status				
Yes	1		_	
No	0.79 (0.46-1.36)	0.410	_	_
Marital status				
Single	1		_	
Married	0.81 (0.48-1.36)	0.431	_	_
HPV/p16 status				
Negative	1		_	
Positive	0.81 (0.46-1.40)	0.451	_	_
Subsite				
Posterior oropharyngeal wall	1			
BOT	1.53 (0.65-3.60)	0.326	_	
Palatine tonsil	1.65 (0.58-4.69)	0.341	_	_
Overall stage			_	_
III	1		_	_
IV	0.57 (0.27-1.21)	0.148	_	_

In the multivariate analysis, variables like gender, and BMI both showed the statistical significance and the LRC risk in gender/female is increased 3 times (HR 2.65; 95% CI, 1.25-5.60; p=0.010) and the patients with overweight BMI (25.0 to 29.9 kg/m^2^) showed almost three times the risk of death (HR 2.76; 95% CI, 1.32-5.76; p=0.007) (Table 3).

Kaplan-Meier curves for overall survival and locoregional control

**Figure 2:**
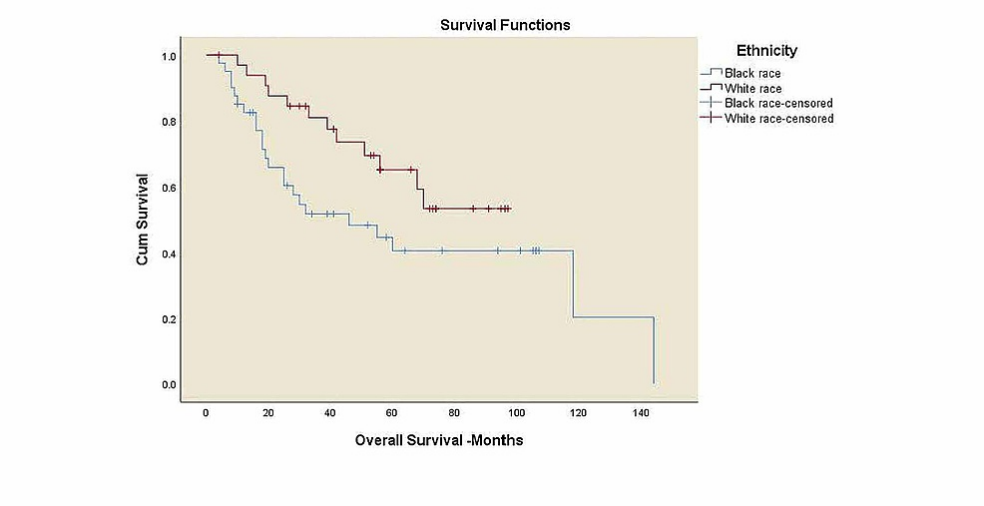
Overall Survival for all patients by race/ethnicity. OS: overall survival. Black race: five-year OS 40.6%, white race: five-year OS 65.3%, p=0.063.

**Figure 3:**
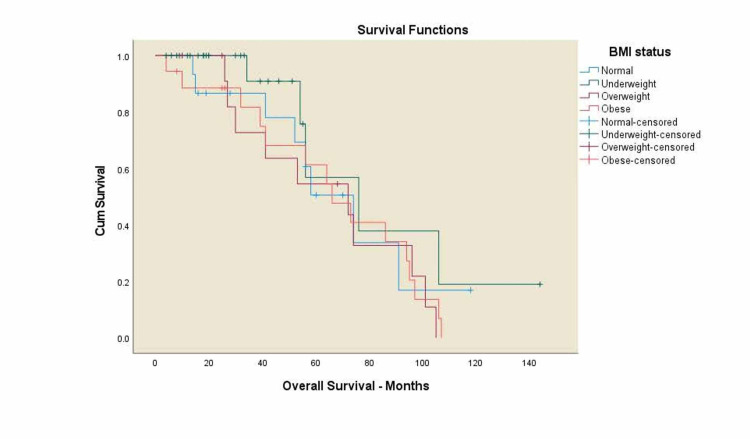
Overall survival for all patients by BMI. BMI: body mass index; OS: overall survival. Normal weight: five-year OS 50.6%, underweight: five-year OS 56.8%, overweight: five-year OS 43.6%, obese: five-year OS 51.3%, p=0.425.

**Figure 4:**
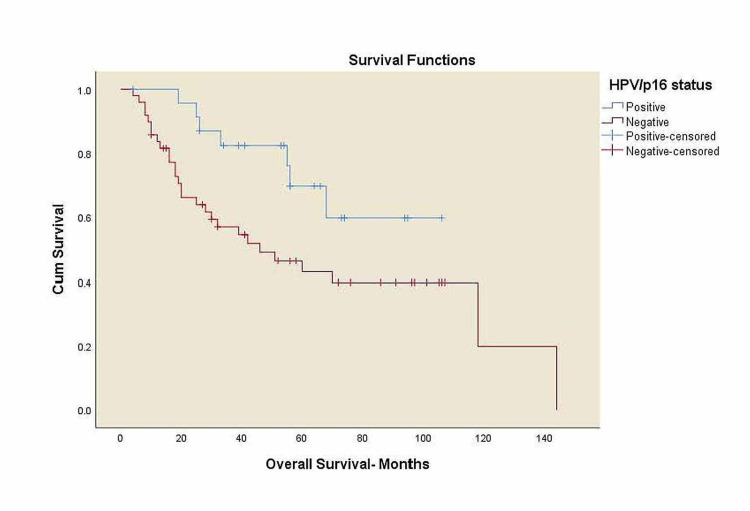
Overall survival for all patients by HPV/p16 status. HPV: human papillomavirus, OS: overall survival. HPV/p16 positive: five-year OS 69.7%, HPV/p16 negative: five-year OS 43%, p=0.034.

**Figure 5:**
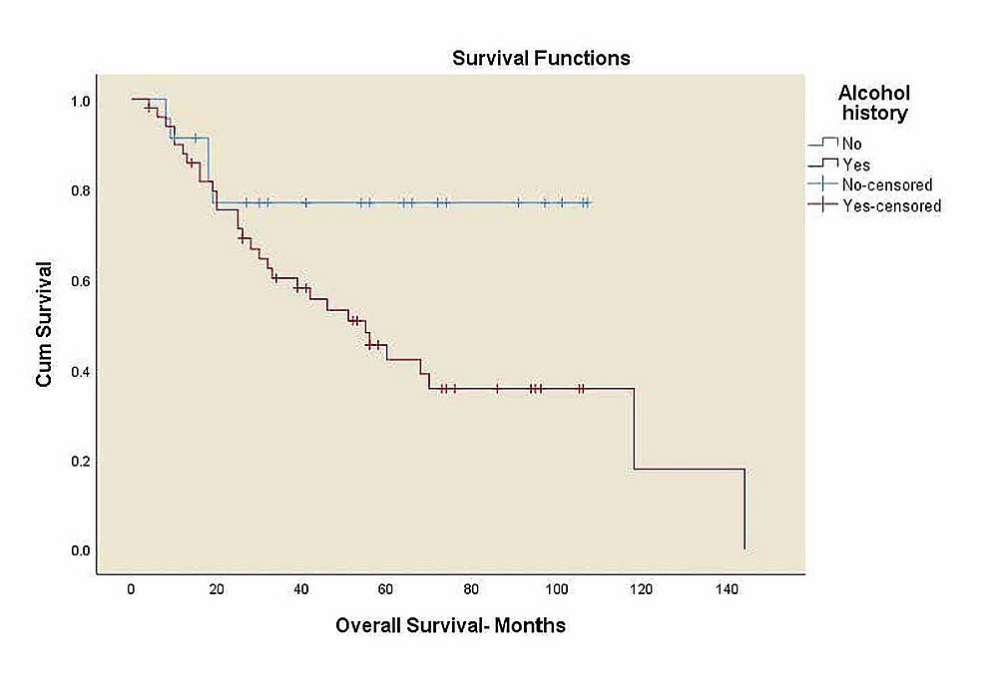
Overall survival for all patients by alcohol history. OS: overall survival, without alcohol history: five-year OS 76.9%, with alcohol history: five-year OS 42.3%, p=0.034.

**Figure 6:**
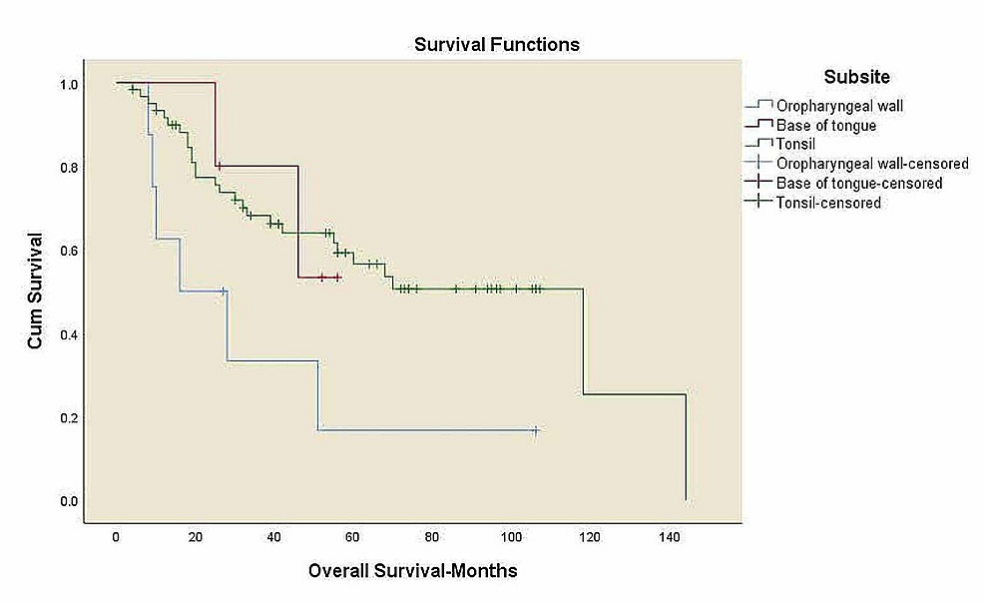
Overall survival for all patients by tumor subsite. OS: overall survival. Oropharyngeal wall: five-year OS 16.7%, base of tongue: five-year OS 53.3%, tonsil: five-year OS 56.5%, p=0.047.

**Figure 7:**
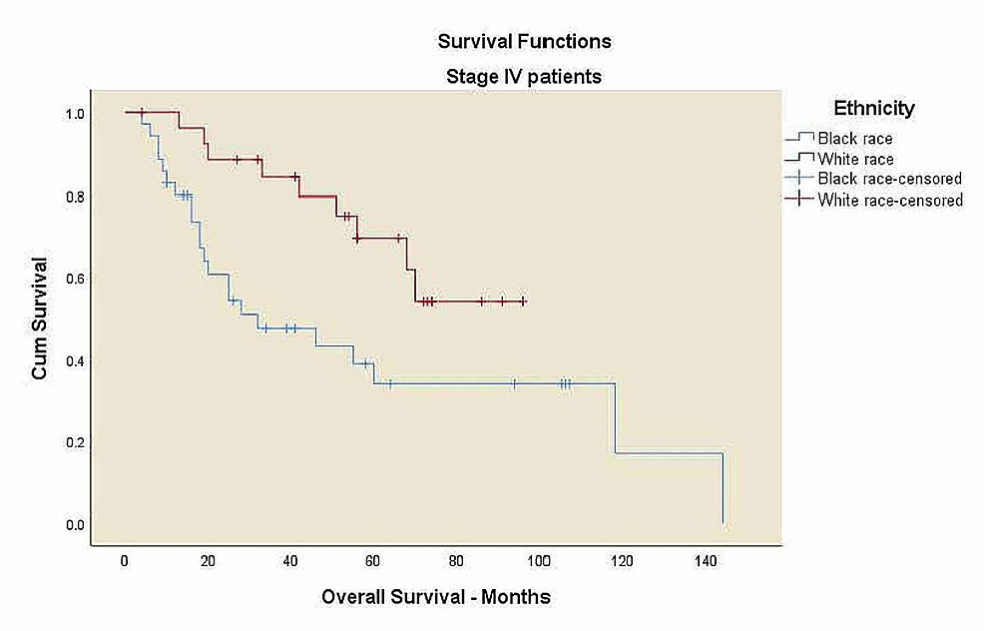
Overall survival for Stage IV patients by ethnicity. OS: overall survival. Black race: five-year OS 34%, white race: five-year OS 69.5%, p=0.014.

**Figure 8:**
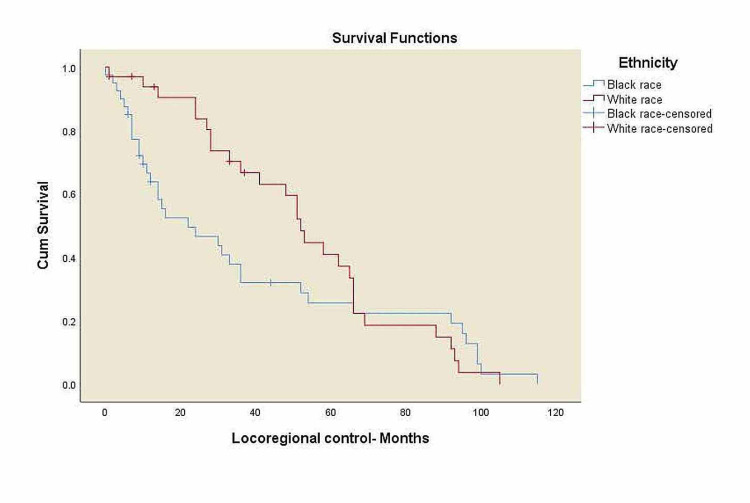
Locoregional control for all patients by race/ethnicity. LRC: locoregional control. Black race: three-year LRC 37.8%, white race: three-year LRC 66.8%, p=0.354.

The p16 negative patients showed statistically significant worse 5-year OS compared to p16 positive patients, 43% vs. 69.7% (p=0.034). Median follow-up for the entire group was 41 months (range: 4-144 months). Kaplan-Meier survival curves were shown in Figures 2-8. The alcohol history, tumor subsites and p16 status, were all independently associated with statistically significant OS rates. For Stage IV, the black patients showed statistically significant worse five-year OS compared to white patients, 34% vs. 69.5% (p=0.014).

There was a trend toward worse OS among black patients compared to white patients, 41% vs. 65% (p=0.063), and decreased survival in overweight patients compared to normal-weight patients, 43.6% vs. 50.6% (p=0.425) with no statistical significance. A significant trend towards decreased survival in patients with alcohol history compared to the patients without alcohol history, 42.3% vs. 76.9% (p=0.034), and a decreased survival rates in p16 negative patients compared to p16 positive patients, 43% vs. 69.7% (p=0.034) was noticed. Three-year LRC for black patients was 37.8% vs. 66.8% in white patients (p=0.354) with no statistical significance.

## Discussion

In this retrospective study of 73 oropharyngeal SCCa patients, we evaluated variables, socio-demographic individual characteristics (race/ethnicity, age, gender, BMI, marital status, health insurance); clinical characteristics (tumor site, tumor grade and p16 status); county-level SES attributes (age distribution, unemployment, income, distance traveled to treatment facility, smoking, and alcohol history).

We found the following important findings.

Our study found no statistically significant difference in survival outcomes between black and white patients’ among Stage III & IV SCCa. This may be due to few numbers of patients in Stage III (15.1% vs. 84.9%) compared to Stage IV. However, racial factor appears to be significant in Stage IV patients, the black patients showed statistically significant worse 5-year OS compared to white patients, 34% vs. 69.5% (p=0.014).

Our study reports that overweight patients show statistically significant increased risk of death compared to normal weight patients, (HR 4.75, 95% CI, 1.31-17.24; p=0.018)

Our study demonstrates that patients with alcohol history show a statistically significant worse survival compared to the patients without alcohol history, 42.3% vs. 76.9% (p=0.034).

Our study observed a trend of statistically significant worse survival for p16 negative patients compared to p16 positive patients, 43% vs. 69.7% (p=0.034).

In our study population, white patients present overall the best distribution according to the patient, tumor, socioeconomic and county-level attributes, similar to previous studies where black patients were more frequently presented with the least favorable distribution [18]. Many studies reported that the association of the black population with lower household income, lower employment rates are more likely associated with increased rates of smoking and heavy alcohol use [19,20]. Our study found no statistical difference in survival outcomes between the black patients and the white patients, because of few numbers of patients in Stage III compared to Stage IV. However, in Stage IV, black patients showed statistically significant worse survivals compared to their white counterparts.

The smoking and alcohol consumption rates were similar between white and black patients; however, in overall cohort, the patients with alcohol history demonstrated worse survival compared to the patients without alcohol history. Black oropharyngeal SCCa patients are more likely to reside in lower education and lower-income level and less likely to have insurance compared with the white study population [19,20-23]. In our study, black patients (62.5%) were reported to be from low-income level compared to their white counterparts (36.4%).

A study conducted by Choi et al. showed that patients with the following characteristics, the race black, low-income level, low educational level, advanced age, being single, smoking history, alcohol history, lower BMI, advanced clinical stage, and treated with chemotherapy were associated with poor prognosis [24,25]. In our study, the results observed for OS univariate analysis by Cox regression showed that having an overweight BMI, alcohol history, p16 negative status and BOT tumor subsite were associated with poor prognosis. However, in the multivariate model, we found that the BMI is the only important prognosis factor. It’s been observed in other studies that black patients were higher at risk for death due to HN cancers. In our cohort, the observed differences with no statistical significance in OS considering race/ethnicity, indicates that equal clinical care (access to care and quality of care) provided at our state academic medical center and SES could bring more equity in racial/ ethnic differences observed for oropharyngeal SCCa survival rates.

Tumors induced with HPV have different biology than tobacco and alcohol-induced tumors [26-28]. HPV/p16 is one of the risk factor for oropharyngeal SCCa, but some studies showed that p16 positive tumors present a better prognosis than tobacco and alcohol-induced oropharynx tumors [29,30]. In our cohort, we observed a trend of worse OS for p16 negative (43%) compared to p16 positive patients (69.7%) which is inexplicable.

In our analysis of LRC, variables like gender, and BMI showed a statistically significant difference in prognosis. We observed that female patients presented a worse prognosis when compared to male patients, the overweight BMI patients presented twice the risk of relapsing oropharyngeal SCCa and p16 negative showed 19% decreased risk of relapse compared to p16 positive patients. In the multivariate analysis, gender and BMI are independent prognosis factors for LRC.

Equal access to healthcare is crucial to reduce health disparities along with lifestyle interventions such as dietary modification, smoking cessation, alcohol cessation and increased exercise should be incorporated into treatment guidelines and considered when defining research priorities.

Limitations

The number of patients in our retrospective study is very small and the income variable in this study is at the census tract level, so there might be a possibility of SES differences at the individual level confounding the racial/ ethnic differences reported.

## Conclusions

Our retrospective study shows that p16 status, tumor subsites, BMI level, and alcohol history play important role in oropharyngeal SCCa survival rate. In the demographic and clinical analysis, the variables income quartile and p16 status shown statistical significance. In the univariate analysis, when adjusted for patients characteristics (BMI and alcohol history), tumor characteristics (p16 status and tumor subsite) showed a higher risk of death from oropharyngeal SCCa for our study population. In the subsequent multivariate analysis, the risk of death from oropharyngeal SCCa was no longer statistically significant for alcohol history, tumor subsite or p16 status but still high for BMI. In this retrospective study, we did not find statistical significant poor outcomes among any specific race, indicating an equal access to healthcare in our state academic medical center. In the future, we need to aim at the individual SES rather than census tract to better understand its effect on the racial disparity. In addition, studies should investigate the mechanisms surrounding the relationships between BMI and risk for HN cancer observed in this study as well as its implications on disease management and prevention.
